# A Rare Presentation of Edwards Syndrome in a Three-Month-Old Infant: A Case Report

**DOI:** 10.7759/cureus.53105

**Published:** 2024-01-28

**Authors:** Anirudh Kommareddy, Jayant D Vagha, Keta Vagha, Amar Taksande, Chaitanya Kumar Javvaji

**Affiliations:** 1 Pediatrics, Jawaharlal Nehru Medical College, Datta Meghe Institute of Higher Education and Research, Wardha, IND

**Keywords:** morbidity, respiratory distress, micrognathia, rocker bottom foot, edwards syndrome

## Abstract

Edwards syndrome, also known as trisomy 18, is a rare chromosomal disorder associated with multiple congenital anomalies and high morbidity. This report presents the case of a three-month-old female infant diagnosed with Edwards syndrome, presenting classic phenotypic features, including low-set ears, micrognathia, and a rocker bottom foot. The infant's condition was further complicated by cardiac abnormalities and respiratory distress, necessitating a comprehensive, multidisciplinary approach involving pediatricians, cardiologists, and orthopedic specialists. The diagnostic journey involved addressing challenges related to respiratory distress syndrome, bronchiolitis, and cardiac complications. The management approach underscored the significance of individualized care tailored to the patient's unique needs. Genetic counseling played a pivotal role in providing essential support to the family facing the complexities associated with Edwards syndrome. This case report highlights the intricacies of Edwards syndrome and contributes to the ongoing discourse on refining clinical strategies for enhanced care and compassionate support. Additionally, it emphasizes the need for further research to advance our understanding of this condition and guide future interventions.

## Introduction

Edwards syndrome, or trisomy 18, is a chromosomal disorder characterized by the presence of an extra copy of chromosome 18, resulting in multiple congenital anomalies and severe intellectual disabilities [1]. It is a relatively rare condition with an estimated prevalence of one in 5,000 live births and is more commonly observed in females than males [1,2]. The syndrome is associated with high morbidity and mortality, with a significant number of affected pregnancies ending in spontaneous abortion or stillbirth [2,3]. The clinical manifestations of Edwards syndrome are diverse and often involve various organ systems. Common phenotypic features include low-set ears, micrognathia, a depressed nasal bridge, clenched fists, and rocker bottom feet [3,4]. Additionally, cardiac abnormalities, such as ventricular septal defects and hypertrophic cardiomyopathy, contribute to the complexity of the syndrome [5].

The neurological and developmental aspects of Edwards syndrome further complicate the clinical picture. Most affected individuals exhibit severe intellectual disabilities, and survival beyond the neonatal period is rare [3,6]. Neonates with Edwards syndrome often face challenges such as feeding difficulties, respiratory distress, and cardiac complications, contributing to the overall high mortality rate [7]. Diagnosing Edwards syndrome involves karyotyping or molecular genetic testing to confirm the presence of an extra chromosome 18 [3]. Prenatal screening and diagnosis have become more prevalent, allowing for early identification and counseling for families facing the challenges associated with this condition [8]. The diagnostic journey involved identifying cardiac abnormalities through a two-dimensional (2D) echocardiogram and managing respiratory distress and bronchiolitis. A comprehensive, multidisciplinary approach was essential to address the complex medical needs of infants with Edwards syndrome. Appreciating the diverse clinical presentations and associated challenges is crucial for healthcare professionals caring for affected individuals. Furthermore, it highlights the significance of providing genetic counseling and support to families confronting a trisomy 18 diagnosis.

## Case presentation

**Figure 1:**
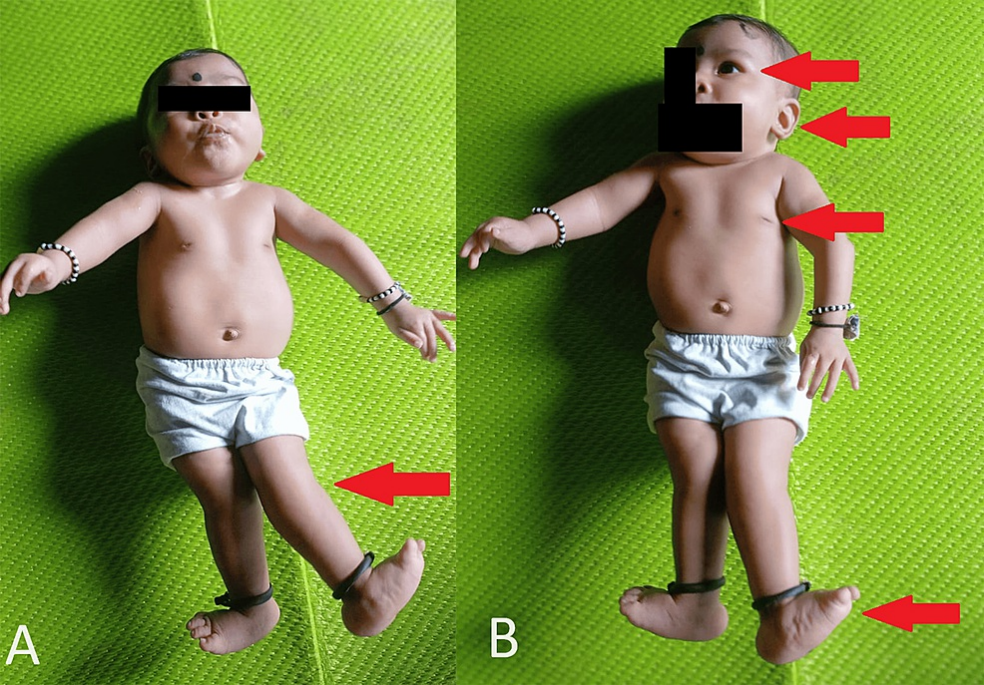
(A) Recurvatum deformity. (B) Depressed nasal bridge, low set ears, widely spaced nipples, and rocker bottom deformity.

A three-month-old female infant was presented by her parents, expressing concerns about a persistent cough and breathlessness over the past 10 days. The mother reported that similar symptoms had been noted since the infant's birth. The infant had previously been admitted to a private hospital in a rural hospital 20 days ago, where she underwent treatment for cough and breathlessness with various medications. Notably, the baby has a medical history of forehead sweating, a suck-rest-suck cycle, and recurrent respiratory tract infections from birth. Born with a weight of 2.5 kg, the infant did not cry immediately after birth and spent 13 days in the neonatal intensive care unit (NICU). A rocker bottom foot was also noted, for which a hip spica cast was applied for a month. The persistent respiratory issues led to a diagnosis of respiratory distress syndrome with bronchiolitis. Subsequently, the infant was referred to a rural hospital, where syndromic features such as low-set ears, micrognathia, a depressed nasal bridge, and widely spaced nipples were observed (Figure 1).

**Figure 2:**
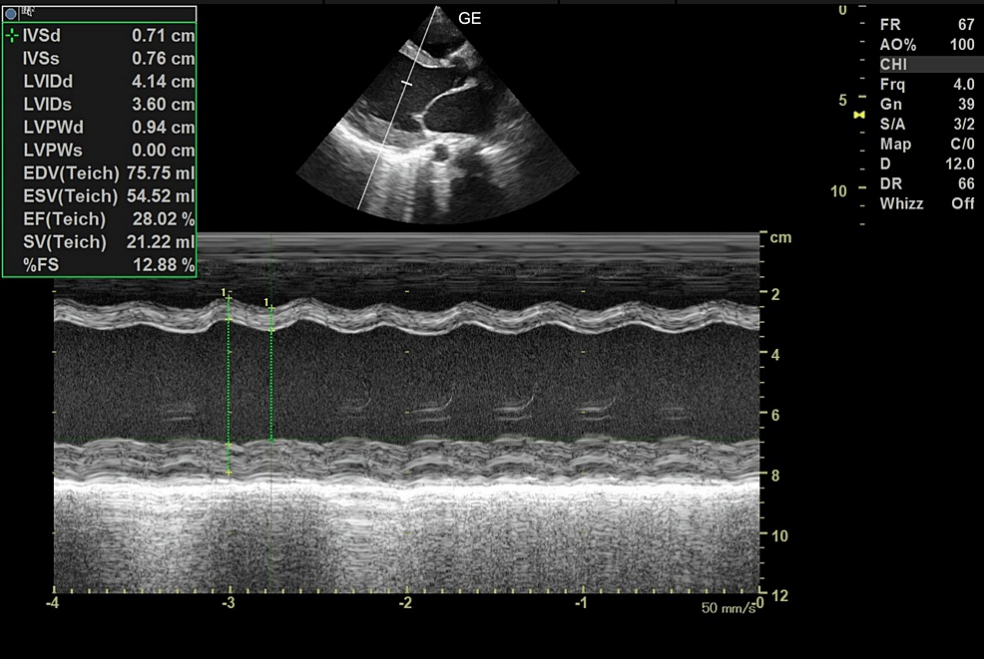
A 2D echocardiogram showing dilated cardiomyopathy with an ejection fraction of 28%.

A 2D echocardiogram revealed dilated cardiomyopathy with an ejection fraction of 28% (Figure 2).

**Figure 3:**
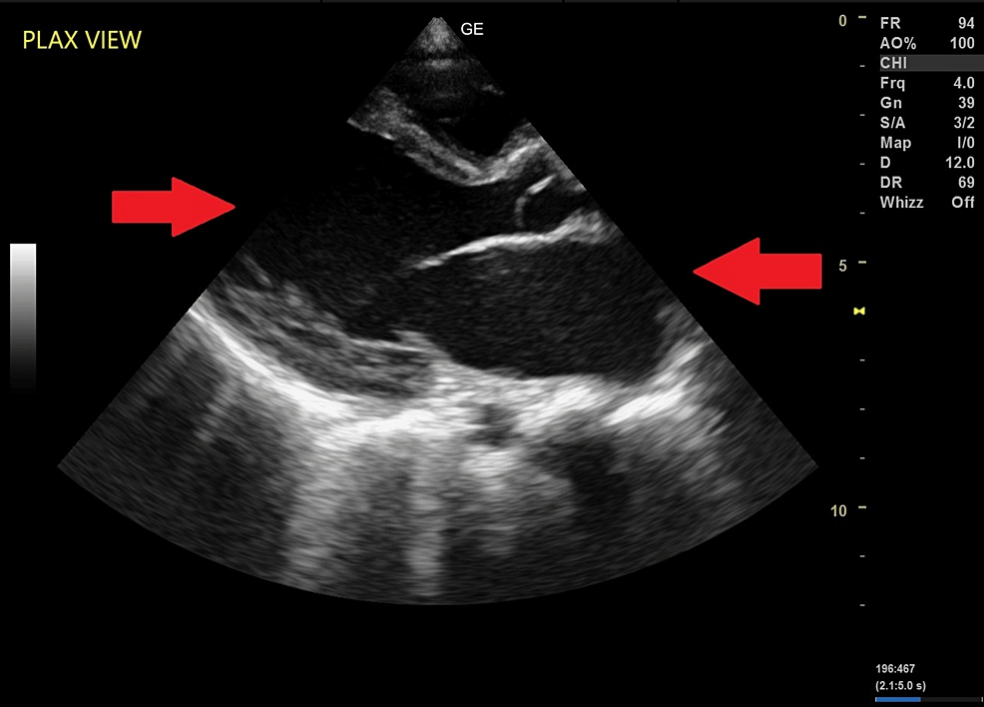
Ultrasound in the PLAX view showing dilated left atrium and left ventricle. PLAX, parasternal long axis

**Figure 4:**
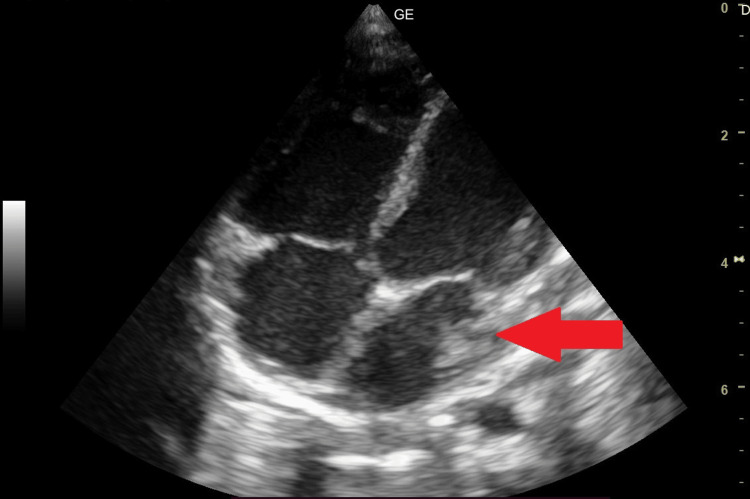
Ultrasound showing a rare association of intracardiac appendage in the left atrium attached to the atrial wall.

Ultrasound in the parasternal long-axis view showed a dilated left atrium and left ventricle (Figure 3) and a rare association of intracardiac appendage in the left atrium attached to the atrial wall (Figure 4).

**Figure 5:**
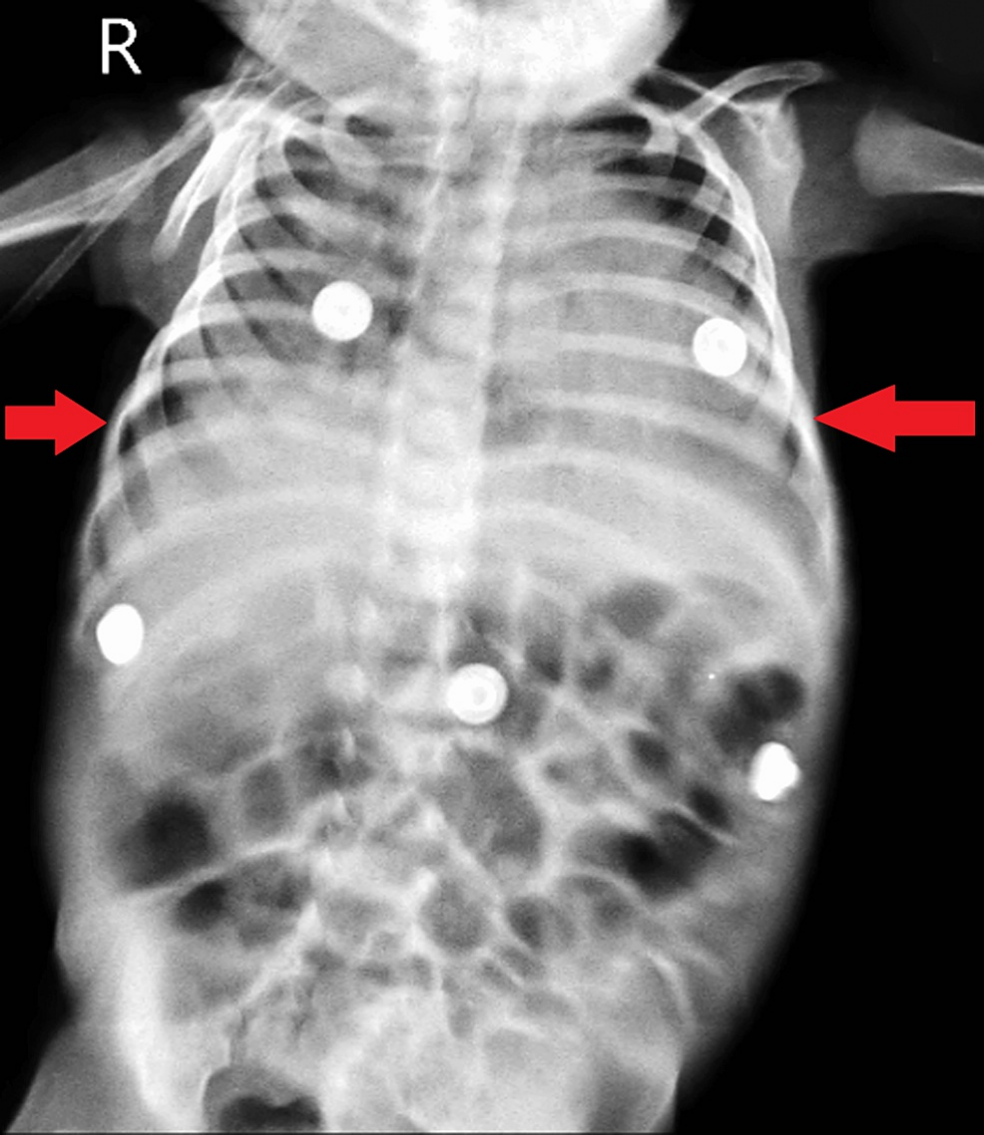
External chest X-ray showing bilateral patches and diffuse haziness on the right side.

An external chest X-ray showed bilateral patches and diffuse haziness on the right side (Figure 5). The infant experienced multiple episodes of loose stools, cough, cold, and fever intermittently since birth, according to the mother. After a 10-day admission to a rural hospital, the infant was referred to a tertiary care hospital for further management.

Upon admission, the infant exhibited bilateral coarse crepitations with no murmur, a distended abdomen with visible engorged veins, and an open anterior fontanelle. Injectable antibiotics and nebulization were initiated, along with continuous positive airway pressure (CPAP) due to persistent distress. Nebulization with Budecort was administered for noisy breathing, and syrup oseltamivir was introduced. The infant maintained saturation on CPAP, and nebulization with ipratropium was added for wheezing. Syrup Deocal was included, and a chest X-ray revealed patchy infiltrates. Following a 2D echocardiogram suggesting biventricular concentric hypertrophy, a normal ultrasound of the abdomen and pelvis was performed.

**Figure 6:**
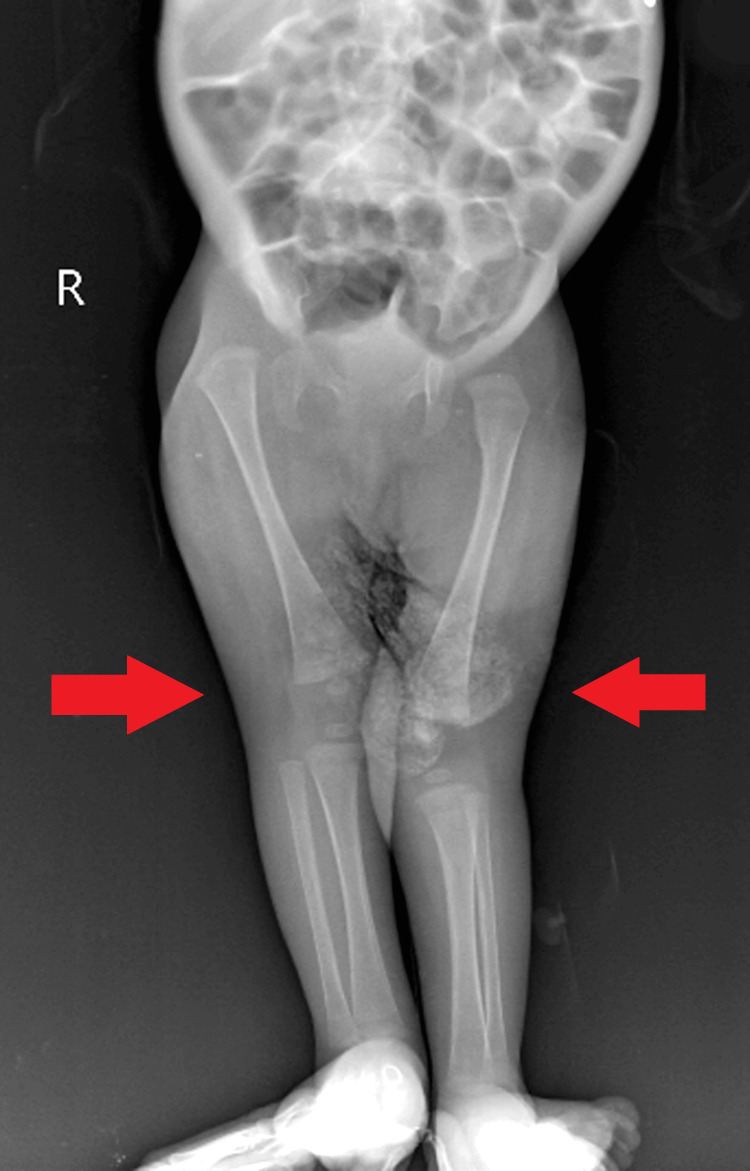
A visual representation of bilateral limb deformity and rocker bottom foot.

An orthopedic consultation was sought for limb deformity and rocker bottom foot (Figure 6), with a recommendation for hip spica under general anesthesia for right-sided developmental dysplasia of the hip (DDH). No active management was advised for the rocker bottom foot at present.

CPAP was discontinued, and the infant maintained saturation on oxygen via nasal prongs, gradually being weaned off. Additionally, due to underlying heart disease and congestive heart failure (CHF), the respiratory distress was exacerbated and prolonged. However, no specific medical treatment for heart failure was mentioned, and laboratory tests for infection, inflammation, renal function, and anemia were not reported. Syrup cefpodoxime was continued for 10 days and then discontinued. Pediatric fitness for hip spica was deferred due to bronchiolitis. The child was discharged with instructions to follow up with bronchoscopy reports. A fiberoptic bronchoscopy was recommended, and the infant was referred to a higher center for the procedure.

## Discussion

Edwards syndrome, also known as trisomy 18, is a chromosomal disorder associated with multiple congenital anomalies and a high rate of mortality, particularly in the neonatal period. This case report highlights the challenges in diagnosing and managing a rare presentation of Edwards syndrome in a three-month-old female infant. The clinical features observed in this case align with the typical manifestations of Edwards syndrome, including low-set ears, micrognathia, a depressed nasal bridge, and widely spaced nipples [2]. These phenotypic characteristics, coupled with the history of respiratory distress syndrome, bronchiolitis, and cardiac abnormalities such as dilated cardiomyopathy, contribute to the clinical suspicion of an underlying genetic disorder [2,9].

The infant's neonatal course, marked by a lack of immediate crying after birth, a prolonged stay in the NICU, and the presence of a rocker bottom foot, further raises suspicion for Edwards syndrome [9]. The association of rocker bottom feet with trisomies, particularly trisomy 18, has been documented in the literature [10]. Cardiac abnormalities, a common feature in Edwards syndrome, were confirmed through a 2D echocardiogram revealing dilated cardiomyopathy [11]. Cardiac involvement in Edwards syndrome is a significant contributor to the morbidity and mortality associated with the condition.

Respiratory symptoms, including persistent cough and breathlessness, compounded the challenges in managing this case. The need for CPAP and bronchiolitis diagnosis underscores the respiratory component of Edwards syndrome, which can present with a spectrum of respiratory issues [6]. The delayed initiation of hip spica due to bronchiolitis reflects the intricacies of managing multiple organ system involvement in these cases. The need for orthopedic intervention for DDH highlights the importance of a multidisciplinary approach in caring for infants with Edwards syndrome. While there is no specific cure for Edwards syndrome, a thorough and accurate diagnosis is crucial for appropriate management and counseling of the affected families. The referral for fiberoptic bronchoscopy indicates the ongoing effort to comprehensively address the respiratory concerns.

## Conclusions

In conclusion, the presented case of a three-month-old female infant with Edwards syndrome underscores the intricate diagnostic and management challenges associated with this uncommon chromosomal disorder. The clinical scenario exhibited typical features such as low-set ears, micrognathia, and a rocker bottom foot, along with notable cardiac complications and respiratory distress. The collaborative efforts of a multidisciplinary healthcare team involving pediatricians, cardiologists, and orthopedic specialists were vital in addressing the diverse medical complexities of the infant. There is currently no cure for Edwards Syndrome; therefore, the emphasis remains on providing comprehensive supportive care tailored to each patient's needs. The case highlights the necessity of genetic counselling for affected families. It underscores the significance of continued research to enhance our understanding of Edwards syndrome, paving the way for improved clinical management and compassionate support. Ultimately, this case contributes to the ongoing dialogue within the medical community on refining strategies for the care and well-being of individuals affected by rare chromosomal disorders.
